# COVID-19 outbreak at an aged-care facility in Selangor, Malaysia, March–April 2020

**DOI:** 10.5365/wpsar.2022.13.1.839

**Published:** 2022-01-25

**Authors:** Faridah Jafri, Mardiana Omar, Faridah Kusnin, Masitah Mohamed

**Affiliations:** a Klang District Health Office, Ministry of Health Malaysia, Putrajaya, Malaysia.

## Abstract

**Objective:**

Aged-care facilities are high-risk settings for coronavirus disease 2019 (COVID-19) outbreaks because residents have risk factors such as advanced age and multiple comorbidities. This report details a COVID-19 outbreak at an aged-care facility in Selangor, Malaysia during March–April 2020.

**Methods:**

Epidemiological and environmental data were gathered via telephone interviews and field investigations. Swab samples were taken from all residents and staff for laboratory investigation. Possible contributing factors to the outbreak were explored.

**Results:**

There were a total of 18 individuals at the institution: nine elderly residents and nine staff. The attack rate was 66.67% (6/9) among the elderly residents and 55.56% (5/9) among the staff. The most common symptoms reported were fever, cough, shortness of breath and diarrhoea. The fatality rate among COVID-19 cases was 18.18% (2/11). Both fatal cases occurred in people of advanced age (86 and 92 years old), who had comorbidities and had fever at presentation. The factors contributing to the outbreak included a delay in isolating symptomatic residents, the use of common facilities, caregivers providing support to more than one resident and a lack of natural ventilation.

**Discussion:**

Prevention and control measures must be aggressively implemented in high-risk sites to significantly reduce the risk of morbidity and mortality during COVID-19 outbreaks. Specific guidelines should be developed detailing the management of outbreaks in institutions such as aged-care facilities.

As of 2 January 2022, there were approximately 289 million confirmed cases of coronavirus disease 2019 (COVID-19) globally, with over 5.4 million deaths. (*1*) Malaysia recorded its first confirmed COVID-19 case on 2 January 2020, and by 6 July 2020, a total of 8476 confirmed cases of COVID-19 were recorded, with 121 deaths. During that period, three clusters involving aged-care facilities were reported in Selangor state, resulting in 44 infections and five deaths. (*2*) The first COVID-19 case that resulted in death involving a resident of an aged-care facility in the Klang district was notified to the Klang District Health Office on 27 March 2020 from a university hospital.

This report details the investigation and measures taken at this aged-care facility in Klang during the outbreak. The aims were (i) to verify the outbreak and cluster; (ii) to identify cases and describe the outbreak in terms of persons, place and time; (iii) to ascertain the outbreak source, classify at-risk groups and risk factors for disease transmission; and (iv) to implement infection prevention and control measures at the facility.

## Methods

The aged-care facility is privately owned, and it opened in Klang district, Selangor, in December 2019. There are nine staff and nine elderly residents. At the time of the outbreak, all residents had pre-existing comorbidities, including hypertension, diabetes mellitus and cardiovascular disease. Three of the residents required special aids for activities of daily living, which included the use of wheelchairs and specially designed beds.

A suspected case was defined as a person who met the clinical or epidemiological criteria. Clinical criteria were acute respiratory symptoms with at least one of the following: shortness of breath or cough or sore throat and/or fever beginning sometime between 9 March and 7 April 2020 (28 days = 2 incubation periods). Epidemiological criteria included residing or working at the facility anytime within the 14 days before symptom onset or from 9 March to 7 April. A confirmed case was an individual with laboratory confirmation of SARS-CoV-2 infection.

Telephone investigations were conducted with the staff of the facility to gain information about residents and staff. All residents and staff were screened as part of active case detection on 30 March. The information collected included demographic data, clinical symptoms and details of close contacts. Oropharyngeal or nasopharyngeal swab samples were taken and analysed by reverse transcription–polymerase chain reaction at the National Public Health Laboratory in Sungai Buloh. Repeat swab samples were also taken at the university hospital and were analysed at the hospital’s laboratory. A field assessment, which investigated the physical aspects of the facility and residents’ social interactions and activities, was conducted at the institution to ascertain possible contributing factors to the outbreak.

## Results

### Epidemiology

On the day of mass screening at the facility, four individuals had already been admitted to the university hospital (one staff member and three elderly residents, one of whom had died). Swab samples were taken from the remaining staff and residents at the facility (a total of 14 individuals). The demographic and clinical details of each resident and staff member are outlined in **Table 1**.

**Table 1:** Demographic information and COVID-19 disease course among residents and staff at an aged-care facility, Klang district, Selangor, Malaysia, 2020

Age	Sex	Ethnicity	Comorbidity	Symptoms	Symptom onset date	Sample dates and results		Admission date	Outcome
						Care facility	University hospital		
**Staff**									
35	Male	Pakistani	–	Cough, myalgia	26.03.2020	30.03.2020 Positive	03.04.2020 Positive	04.04.2020	General ward
Unknown	Female	Indian	–	Fever, cough	28.03.2020	30.03.2020 Positive	01.04.2020 Positive	01.04.2020	General ward
36	Female	Chinese	–	Fever, myalgia	28.03.2020	–	29.03.2020 Positive	29.03.2020	General ward
33	Female	Chinese	–	Fever, cough, sore throat	02.04.2020	30.03.2020 Negative	03.04.2020 Negative,	08.04.2020	General ward
							07.04.2020 Positive		
26	Female	Indian	–	Asymptomatic	–	30.03.2020 Negative	03.04.2020 Positive	04.04.2020	General ward
20	Female	Unknown	–	None	–	30.03.2020 Negative	–	–	Home quarantine
30	Female	Malay	–	None	–	30.03.2020 Negative	–	–	Home quarantine
33	Male	Chinese	–	None	–	30.03.2020 Negative	–	–	Home quarantine
36	Male	Chinese	–	None	–	30.03.2020 Negative	–	–	Home quarantine
**Residents**									
92	Female	Chinese	Hypertension, CVD	Fever, shortness of breath, diarrhoea	23.03.2020	–	27.03.2020 Positive	25.03.2020	Deceased (28.03.2020)
85	Female	Chinese	DM, hypertension	Fever, cough	28.03.2020	–	29.03.2020 Positive	29.03.2020	General ward
92	Female	Chinese	DM, hypertension	Cough, shortness of breath	28.03.2020	–	29.03.2020 Positive	29.03.2020	General ward
85	Female	Chinese	DM, hypertension, CKD	Cough, diarrhoea	30.03.2020	30.03.2020 Positive	05.04.2020 Positive	05.04.2020	General ward
86	Male	Chinese	Hypertension, CVD	Fever, cough	01.04.2020	30.03.2020 Negative	01.04.2020 Positive	01.04.2020	Deceased (16.04.2020)
85	Female	Chinese	DM	Asymptomatic	–	30.03.2020 Negative	05.04.2020 Positive	05.04.2020	General ward
67	Male	Chinese	DM, hypertension, prostate disease, CVD	None	–	30.03.2020 Negative	05.04.2020 Negative	05.04.2020	General ward
84	Male	Chinese	DM	None	–	30.03.2020 Negative	05.04.2020 Negative	05.04.2020	General ward
85	Female	Chinese	DM, hypertension, CKD	None	–	30.03.2020 Negative	05.04.2020 Negative	05.04.2020	General ward

The last case involved a 33-year-old female staff member with onset of fever and sore throat on 2 April. All residents and staff of the facility were at the facility during the outbreak period, which may have led to cross-infection during care work, therapy sessions and daily activities.

The attack rate was 66.67% (6/9) among the residents and 55.56% (5/9) among the staff. The fatality rate among cases who tested positive for COVID-19 was 18.18% (2/11). The most common symptoms reported were fever, cough, shortness of breath and diarrhoea. The onset of symptoms for the index case was on 23 March, and onset for the last case was on 2 April. All confirmed cases were admitted to the university hospital ward for treatment and isolation. The three elderly residents with negative COVID-19 results were admitted to the university hospital ward for close monitoring and quarantine. Common features among both cases who died were advanced age (86 and 92 years old), the presence of comorbidities, fever at presentation and admission to the ward within 2 days after symptom onset.

**Fig. 1:**
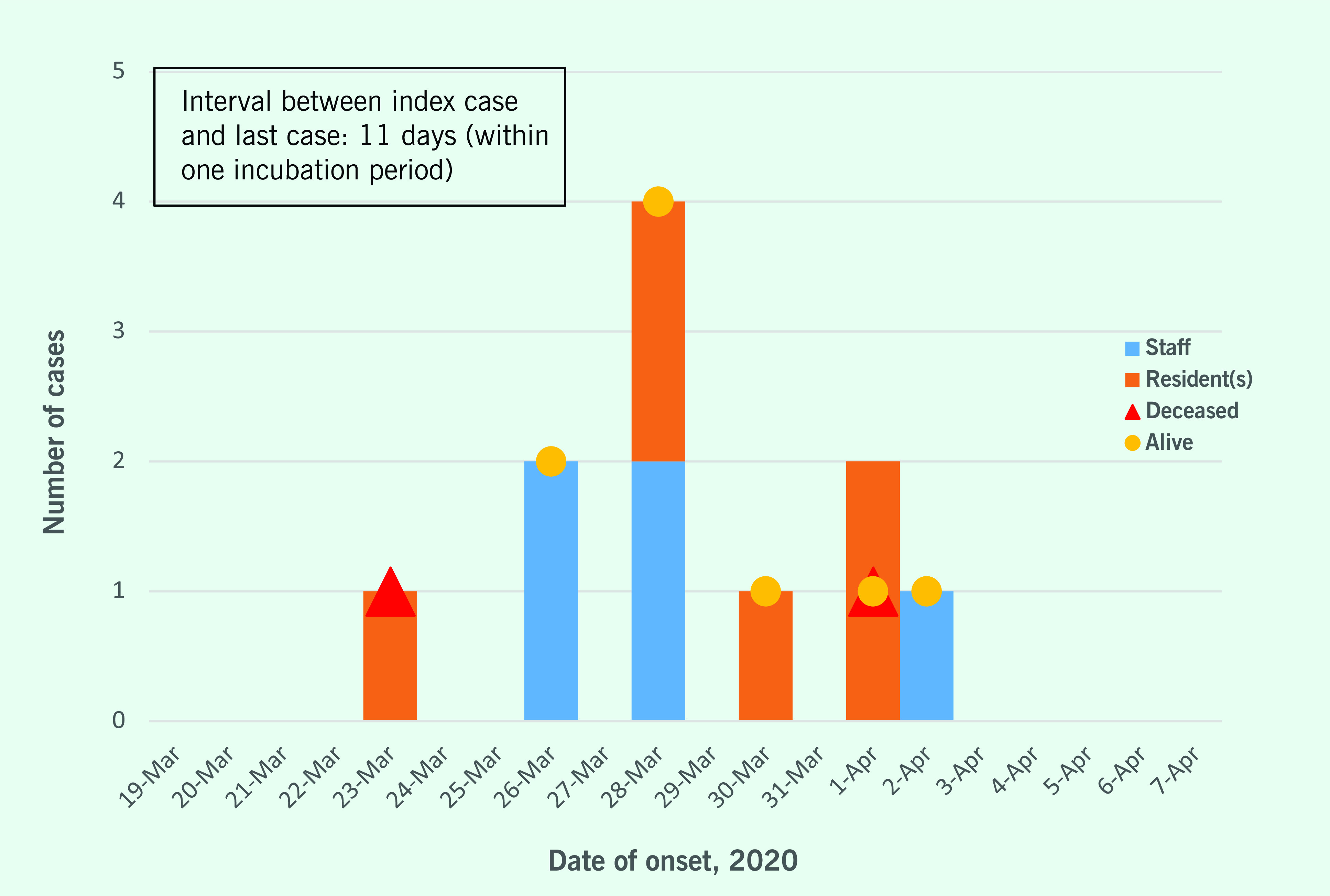
Epidemic curve of the COVID-19 outbreak at an aged-care facility, Klang district, Selangor, Malaysia, 2020

The epidemic curve shows a point source outbreak (**Fig. 1**). The index case was a 92-year-old female with symptoms of fever, shortness of breath and diarrhoea. She had been to hospital previously for anaemia from 7–17 March. She was then discharged to the facility, where she developed symptoms of fever and diarrhoea on 23 March. She was readmitted to hospital on 25 March, but she developed shortness of breath and died 3 days later (**Table 1**).

[**insert** Figure 1]

### Laboratory investigation

There were 14 swab samples taken at the facility on 30 March, three of which tested positive for COVID-19. Fifteen swab samples were taken at the university hospital between 27 March and 7 April (**Table 1**). In total, there were 11 positive cases.

### Environmental investigation

The facility is a two-storey bungalow with five bedrooms and four bathrooms, with a total area of about 353 m^2^ (3800 square feet). There were three double-occupant rooms and two four-occupant rooms for the residents. All rooms had two occupants, except one double room with only one occupant. Common areas included a lounge, a dining room and a kitchen. Outside of the building is an open space used for physical activities. Air conditioning is used constantly at the facility, and windows generally remain closed. Activities conducted individually included personal care, regular health check-ups and physiotherapy sessions. However, caregivers and the physiotherapist attended to multiple residents. Group activities included meal times, exercise and social activities.

The facility’s management staff implemented twice-daily general cleaning and disinfection when the Malaysian government enacted the first Movement Control Order on 18 March 2020. Based on observations and interviews, staff used personal protective equipment inconsistently throughout the outbreak. In general, the level of cleanliness was satisfactory, and measures for physical distancing were in place.

### Infection prevention and control

Health education was delivered to the facility’s management about infection prevention and control measures for COVID-19 outbreaks on 30 March. Total disinfection of the facility was carried out by the municipality on 6 April. The facility was temporarily closed and all residents and staff were issued quarantine orders in an effort to break the chain of transmission and assist in contact tracing. All remaining residents were pre-emptively admitted to hospital on 6 April for close monitoring and quarantine (before the release of swab test results).

## Discussion

The index case of this outbreak was an elderly resident with symptom onset on 23 March 2020. She exhibited symptoms of fever and diarrhoea at the beginning of the infectious period, which subsequently led to hospital admission. The source of her infection was believed to be nosocomial, and infection was believed to have occurred during her previous hospital admission in early March. The week that she stayed at the aged-care facility between the two hospital admissions provided ample opportunity for transmission to take place. The onset of the last case was on 2 April (within one incubation period of the index case); this case had symptoms of fever, cough and sore throat. The time of onset showed that the outbreak was limited because no new cases were reported that exceeded one incubation period.

A few mechanisms may have prompted SARS-CoV-2 transmission at the facility. First, SARS-CoV-2 is transmissible even while a case is asymptomatic or presymptomatic. (*3*) This phenomenon has complicated efforts to isolate infected individuals. Evidence shows that asymptomatic individuals may be infectious as early as 12.3 days (95% confidence interval: 5.9–17 days) before symptom onset. (*4*) Second, the residents’ demographic factors, such as older age and the presence of comorbidities, predisposed them to greater risk of severe infection, with complications and death. (*5*) Third, the shared use of rooms and bathrooms, group activities and social interactions at the facility may have precipitated the spread of infection among the residents. Fourth, the facility was fully air-conditioned, which hindered natural ventilation, thus predisposing residents and staff to the spread of airborne infection. (*6*)

Based on the experiences in this outbreak, we have outlined a few recommendations for improvements to outbreak prevention and control measures in similar facilities. First, upon receiving an outbreak notification, a rapid assessment team should conduct a thorough risk assessment of the facility and its occupants (both residents and staff). Second, a high index of suspicion should be adopted to identify symptomatic positive cases early and isolate them from the rest of the residents and staff. Third, hospital admission should be considered early for elderly residents because they are at great risk for rapid, unpredictable deterioration from SARS-CoV-2 infection. Fourth, repeat testing should be considered in view of the possibility of continuous exposure to asymptomatic cases. Finally, the use of natural ventilation should be encouraged, especially during the day, and windows and doors should be regularly opened at the facility.

Among the limitations of this outbreak investigation were the small sample size and lack of completeness in patient data because they were gathered through telephone interviews with third parties, that is, management staff. Additionally, an outbreak transmission tree could be established through molecular sequencing to better explain the chronology of the outbreak.

## Conclusions

COVID-19 outbreaks at aged-care facilities are serious events, as residents are at high risk of morbidity and mortality. In the outbreak at the aged-care facility in Klang district, health authorities took appropriate measures by conducting mass screening at the facility and isolating elderly residents in the hospital. Specific guidelines for managing institutional COVID-19 outbreaks, such as those occurring at aged-care facilities, should be prepared by ministries of health and other appropriate agencies.

## References

[1] Weekly epidemiological update on COVID-19 – 6 January 2022. Geneva: World Health Organization; 2022. Available from: https://www.who.int/publications/m/item/weekly-epidemiological-update-on-covid-19 — 6-january-2022, accessed 7 January 2022.

[2] Hasmuk K, Sallehuddin H, Tan MP, Cheah WK, Ibrahim R, Chai ST. The long term care COVID-19 situation in Malaysia. London: International Long-Term Care Policy Network; 2020. [cited 2021 September 28]. Available from: Available from https://ltccovid.org/wp-content/uploads/2020/10/Malaysia-LTC-COVID-situation-report-2-October-2020-1.pdf

[3] Ye F, Xu S, Rong Z, Xu R, Liu X, Deng P, et al. Delivery of infection from asymptomatic carriers of COVID-19 in a familial cluster. Int J Infect Dis. 2020 May;94:133–8. 10.1016/j.ijid.2020.03.042 32247826 PMC7129961

[4] He X, Lau EHY, Wu P, Deng X, Wang J, Hao X, et al. Temporal dynamics in viral shedding and transmissibility of COVID-19. Nat Med. 2020 05;26(5):672–5. 10.1038/s41591-020-0869-5 32296168

[5] Liu K, Chen Y, Lin R, Han K. Clinical features of COVID-19 in elderly patients: A comparison with young and middle-aged patients. J Infect. 2020 06;80(6):e14–8. 10.1016/j.jinf.2020.03.005 32171866 PMC7102640

[6] Natural ventilation for infection control in health-care settings. Geneva: World Health Organization; 2009. Available from: https://apps.who.int/iris/handle/10665/44167, accessed 28 September 2021. 23762969

